# miR-876 Inhibits EMT and Liver Fibrosis via POSTN to Suppress Metastasis in Hepatocellular Carcinoma

**DOI:** 10.1155/2020/1964219

**Published:** 2020-10-05

**Authors:** Kai Chen, Zhonghu Li, Mengyun Zhang, Bo Wang, Tao Peng, Yanbing Shen, Jianxin Zhang, Jiaxin Ye, Yu Liu, Di Tang, Minjie Peng, Dandan Ma, Zhengkang Xiao, Yujun Zhang, Weidong Jin, Xiaowu Li

**Affiliations:** ^1^Hepatobiliary Surgery Institute, Southwest Hospital, Army Medical University, China; ^2^Department Hepatobiliary Surgery Institute, Chengdu Fifth People's Hospital, China; ^3^Department General Surgery, Central Theater Command General Hospital of PLA, China; ^4^Department Rheumatology of Integrated Traditional Chinese and Western Medicine, Central Theater Command General Hospital of PLA, China; ^5^Department of Hepatobiliary Surgery, The First Affiliated Hospital of Yangtze University, Jingzhou, China; ^6^Hepatobiliary Surgery & Carson International Cancer Shenzhen University General Hospital & Shenzhen University Clinical Medical Academy Center, Shenzhen University, China

## Abstract

**Background:**

The asymptomatic onset, frequent recurrence, and poor prognosis of hepatocellular carcinoma (HCC) prompted us to identify new therapeutic targets or predictive markers of HCC diagnosis or prognosis.

**Methods:**

In this study, bioinformatics analysis was used to screen for target miRNAs from the open-access TCGA database. Transwell assays, Western blotting, and qRT-PCR analyses were used to detect cellular functions and gene expression in HCC cells and samples. A nude mouse tumorigenesis model was established to facilitate the observation of HCC progression. Other assays included luciferase reporter assays, IHC, and survival analysis.

**Results:**

We found that the identified miR-876 from TCGA was expressed at low levels in HCC cell lines and that low miR-876 expression was corrected with liver cirrhosis, tumor thrombus, and TNM stage. Further research revealed that miR-876 regulated cell invasion, EMT, and collagen expression by targeting POSTN expression. miR-876 and POSTN were inversely correlated in HCC samples and associated with EMT status and liver fibrosis in clinical HCC tissues. miR-876 inhibited the liver cancer progression in *in vivo* animal assays. Finally, both miR-876 and POSTN were risk factors for HCC survival, and HCC patients with combined low miR-876 and high POSTN expression had worse prognosis.

**Conclusions:**

miR-876 inhibited HCC EMT and fibrosis by targeting POSTN, thus affecting HCC progression and prognosis. miR-876 and POSTN may be useful therapeutic targets or prognostic markers of HCC.

## 1. Introduction

Hepatocellular carcinoma (HCC) is one of the most common malignancies and ranks as the second most deadly cancer worldwide [1]. Over the last several decades, numerous interventional therapies, such as hepatic segmentectomy, liver transplantation, transcatheter hepatic arterial chemoembolization (TACE), and radiofrequency ablation, have made great progress, yet patient prognosis is far from satisfactory, with a 5-year survival rate of less than 20% [2, 3]. Asymptomatic onset, rapid tumor progression, metastasis, and recurrence are the main reasons for the poor prognosis [4, 5]. Thus, elucidating the molecular processes and mechanisms underlying tumor progression and developing novel diagnostic or curative treatments are essential for the systematic treatment of HCC.

microRNAs (miRNAs) are a class of evolutionarily conserved small noncoding RNAs that are composed of ~22 nucleotides and can bind to the 3′ untranslated regions (3′ UTRs) of their target messenger RNAs (mRNAs) to either degrade the target gene or inhibit its translation, thus providing posttranscriptional regulation [6, 7]. Studies indicate that miRNAs participate in nearly all processes of tumor biology, including tumor stemness, proliferation, apoptosis, invasion, and metastasis [8]. Emerging studies have also shown that aberrant miRNA expression is involved in various biological behaviors of tumors, including HCC [9, 10]. For instance, miR-876 was downregulated in cholangiocarcinoma (CCA) and inhibited cell growth and apoptosis by suppressing BCL-XL expression [11]. On the other hand, miR-876 was found to inhibit both cell proliferation and metastasis via targeting DNMT3A [12]. However, the identification of key diagnostic and prognostic miRNAs and elucidating the exact underlying molecular mechanism are urgently needed in HCC.

Periostin (POSTN), also known as osteoblast-specific factor 2, is a 93.3 kDa extracellular matrix (ECM) protein [13]. POSTN plays an important role in ECM remodeling by interacting with other proteins, such as fibronectin, tenascin-C, and collagen [14]. It also has essential effects on collagen fibrillogenesis, cell adhesion, wound healing, and EMT [15]. Aberrant POSTN expression has been found in multitude tumor entities, including head and neck, lung, neuroblastoma, breast, and colorectal cancers and HCC, for its roles in cell survival, EMT, angiogenesis, and tumor microenvironment construction [13, 14, 16]. For example, pancreatic cancer cells stimulate stromal cells to secrete POSTN, which induces tumor desmoplasia and EMT to promote tumor progression [17]. However, studies of the expression and regulatory mechanism of POSTN in HCC are relatively rare and urgently needed.

In this study, we identified miR-876 from the open-access, public TCGA database, and the cellular functions and regulation of POSTN were determined. Detailed expression levels of miR-876 and POSTN in clinical HCC samples were detected. Our results indicated that miR-876 and POSTN play a valuable role in HCC and may provide a potential diagnostic marker or therapeutic target for HCC.

## 2. Materials and Methods

### 2.1. Cell Culture and Transfection

The HCC cell line Hep G2, human normal hepatocyte cell line (L02), and HEK-293 were purchased from ATCC; HCC cell lines SMMC-7721, Huh-7, and HCC-LM3 and human astrocyte cell line LX-2 were purchased from the Institute of Biochemistry and Cell Biology (Chinese Academy of Sciences, Shanghai, China). HCC cells and HEK-293 were cultured in DMEM supplemented with 10% fetal bovine serum (FBS) (Gibco, USA) at 37°C in a humidified atmosphere containing 5% CO_2_.

Cell transfections were carried out as described previously [18]. Briefly, for lentivirus transduction, 10^5^ HCC cells were incubated in a 6-well plate with 2 ml of medium containing 100 *μ*l (10^7^ U) of lentivirus particles and 5 *μ*g/ml polybrene for 24 h. Plasmid and siRNA transfections were performed using Lipofectamine 3000 (Invitrogen, USA) according to the manufacturer's instructions. miRNA mimics or scramble control RNAs (RiboBio, China) were transfected into cells at a final concentration of 100 nM using a riboFECTTM CP Kit (RiboBio, China) according to the manufacturer's instructions.

### 2.2. Bioinformatics Analysis

Hepatocellular carcinoma miRNA chip analysis primary data were accessed at The Cancer Genome Atlas (TCGA) public database (http://www.cancergenome.nih.gov/dataportal). The HCC miRNA expression data were downloaded using the SangerBox software (http://sangerbox.com). TCGA ID is listed in Table S1. miRNA expression data from a total of 49 pairs of tumors and the matched adjacent tissues were analyzed, and the top 20 differentially expressed miRNAs are presented in Table 1.

### 2.3. Cell Invasion Assay

HCC cells in 300 *μ*l of serum-free medium were cultured in a chamber containing an 8 *μ*m polycarbonate filter (Millipore, USA) coated with 30 *μ*l of Matrigel (BD, USA), After incubating for 36 h, cells remaining on the upper membrane were removed with a cotton swab and cells that had penetrated the membrane were fixed with 4% formaldehyde and then stained with 0.5% crystal violet for 20 min. All the statistical results were obtained from three independent experiments averaged from five randomly selected image fields.

### 2.4. RNA Isolation and qRT-PCR Analysis

Total RNA was isolated using TRIzol reagent (Thermo Fisher Scientific, USA) according to the instructions provided. First-strand cDNA was generated with a PrimeScript RT Reagent Kit with gDNA Eraser (TaKaRa, Japan), and miRNA reverse transcription was performed using a Mir-X miRNA qRT-PCR SYBR Kit (Clontech, Japan). Real-time PCR was performed using the PrimeScript RT Reagent Kit and SYBR Premix Ex Taq (TaKaRa, Japan) on a CFX96 Real-Time System (Bio-Rad, USA) with the reaction conditions provided in the instructions. The primer details used in the study were included in Table S2. Additionally, the common miRNA mRQ 3′ primer was provided in the Mir-X miRNA qRT-PCR SYBR Kit (Clontech, Japan).

### 2.5. Western Blot Analysis

Total protein of HCC cells was extracted by RIPA lysis buffer (Thermo Fisher Scientific, USA) containing protease inhibitor cocktail tablets (Roche, USA). After measured by a BCA Protein Assay Kit (Beyotime, China), equal amounts of protein (30 *μ*g) were used to SDS-PAGE on 10% polyacrylamide gels and transferred to PVDF membranes (Millipore, USA), which were blocked and blotted with primary antibodies overnight at 4°C. The antibodies used in this study included the following: anti-E-cadherin (1 : 800, 20874-1-AP, Proteintech, USA), anti-N-cadherin (1 : 500, 22018-1-AP, Proteintech, USA), anti-vimentin (1 : 1000, 10366-1-AP, Proteintech, USA), anti-*α*-SMA (1 : 1000, ab124964, Abcam, USA), anti-collagen-I (1 : 1000, 14695-1-AP, Proteintech, USA), anti-POSTN (1 : 800, PA5-34641, Thermo Fisher Scientific, USA), and anti-*β*-actin (1 : 5000, 20536-1-AP, Proteintech, USA). The membranes were washed with PBST and incubated with horseradish peroxidase-conjugated secondary antibody for 2 h, and the immunocomplexes were then visualized using a New Super ECL Detection Kit (KeyGEN BioTECH, China) according to the manufacturer's protocol.

### 2.6. HCC Patients and Clinical Samples

A total of 127 patients underwent surgical resection of primary, pathologically confirmed HCC at the Institute of Hepatobiliary Surgery, Southwest Hospital, Army Medical University, from January 2006 to January 2010. All patients were confirmed to have HCC by ultrasonography, contrast-enhanced CT, or MRI examination and a blood test for AFP. For the clinical characteristics of these patients, please refer to Table 2. A total of 127 fresh frozen tissues were used for RNA isolation; fresh tissues were put into liquid nitrogen immediately after tumor excision and then transferred to -80°C for future use. 80 formalin-fixed, paraffin-embedded tumor specimens were used for immunohistochemistry (IHC), and 127 frozen tumor specimens were used for RNA extraction. All of the patients were followed at least for 5 years. All patients were followed up by radiography, ultrasonography, or CT examination every 3 months after discharge and were followed up monthly by telephone in the clinical follow-up center of the Department of Hepatobiliary Surgery Institute, Southwest Hospital. This study was approved by the Ethics Committee of Southwest Hospital, and all patients provided written informed consent.

### 2.7. Dual-Luciferase Reporter Assay

5 × 10^3^ HEK-293 cells were cultured in a white 96-well plate and then transfected with pGL3 POSTN or pGL3 mut-POSTN plasmid (Sangon Biotech, China) and 8 ng of the internal control pRL-TK Renilla luciferase plasmid (Promega, USA), together with miR-876 (RiboBio, China) at a final concentration of 0, 50, or 150 nM. After a 48 h incubation, the cells were harvested and processed with the Dual-Luciferase Reporter Assay System (E1910, Promega, USA) according to the manufacturer's protocol. The results were quantified as the ratio of firefly luciferase activity/Renilla luciferase activity in each well.

### 2.8. Animal Experiment

All animal experiments were approved by the Institutional Animal Care and Use Committee of Southwest Hospital, Chongqing, China. Briefly, Four- to six-week-old male randomly selected athymic nude mice were obtained from Southwest Hospital (Chongqing, China) and housed in the standard pathogen-free conditions of Southwest Hospital (Chongqing, China). The mice were anesthetized by an intraperitoneal injection of 1% pentobarbital sodium (50 mg/kg). A median abdominal incision was made to expose the liver, and 5 × 10^6^ PDAC cells (suspended in 50 *μ*l of PBS and Matrigel matrix in case of leakage) were injected to the liver. After closing the abdomen, the mice were imaged by the IVIS Lumina II system (Caliper Life Sciences, USA).

### 2.9. Immunohistochemistry

A total of 80 HCC specimens were fixed in formalin, embedded in paraffin, and cut into 3-micrometer serial sections. The slides were deparaffinized, treated with 10% goat serum to block endogenous peroxidase activity, and heated in 10 mM citrate buffer at 120°C for 2 min and 10 sec for antigen retrieval. Each section was incubated with the following primary antibodies at 4°C overnight: anti-E-cadherin (1 : 200, 20874-1-AP, Proteintech, USA), anti-N-cadherin (1 : 200, 22018-1-AP, Proteintech, USA), anti-vimentin (1 : 200, 10366-1-AP, Proteintech, USA), and anti-POSTN (1 : 400, PA5-34641, Thermo Fisher Scientific USA). Then, the samples were incubated with a secondary peroxidase-conjugated antibody for 60 min at 37°C and then developed with DAB (Dako, 00080066).

The procedures of IHC score were carried out as described previously [19]. Briefly, ten random fields were selected, and expression was evaluated in 1000 tumor cells (100 cells per field) with an image analyzer (MetaMorph Imaging System version 6.0); then, the slides were scored as 0, 1, 2, 3, and 4 if the percentages of positive cells were less than 5%, 6-25%, 26-50%, 51-75%, and 76-100%, respectively. The staining intensity of each antibody was scored as 0, 1, 2, and 3 according to the intensity of positive staining color. Then, the final score was evaluated by the multiplications of the scores of positive cells and intensity of positive staining: 1+ (multiplication 1-4), 2+ (multiplication 5-8), and 3+ (multiplication 9-12). The immunohistochemical scores were further grouped into the following two categories: low (grade 0 or 1+) or high (grade 2+ or 3+).

### 2.10. Statistical Analysis

The correlation between clinical categorical parameters and miR-876 or POSTN expression (the median was regarded as the cutoff value) was evaluated by a *χ*^2^ test. Student's *t*-test was used to compare group differences if they followed a normal distribution; otherwise, the nonparametric Mann-Whitney test was adopted. One-way ANOVA was applied to compare the differences among 3 groups. For survival analysis, univariate analysis was conducted by the KM method (the log-rank test), and multivariate analysis was performed by the stepwise Cox multivariate proportional hazard regression model (Forward LR, likelihood ratio). All the *in vitro* experiments were replicated three times. All analyses were performed using SPSS 26.0 software (IBM, USA), all the tests are two-sided, and a *P* value < 0.05 was considered to be statistically significant.

## 3. Results

### 3.1. The Identified miR-876 Was Expressed at Low Levels in HCC Cell Lines

**Figure 1:**
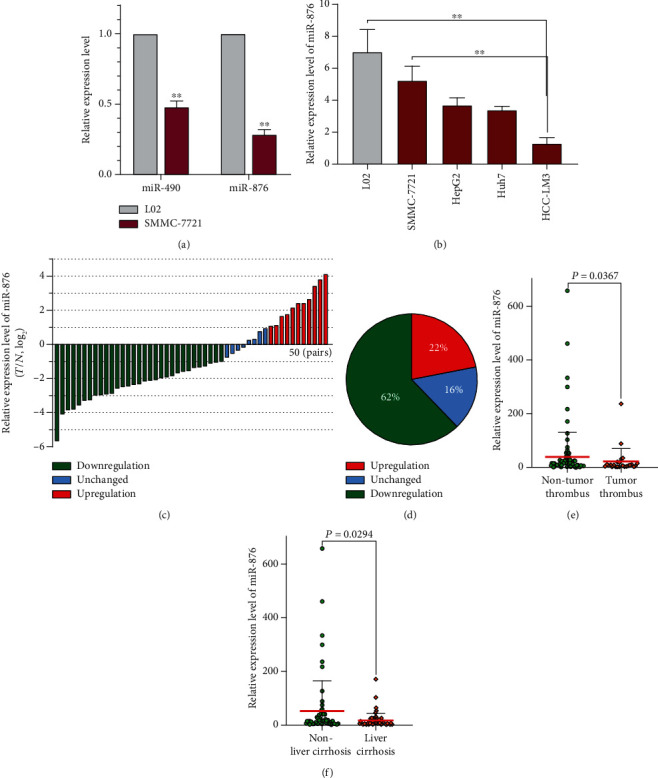
The identified miR-876 was low in HCC. (a) The expressions of miR-490 or miR-876 were detected in indicated cells by qRT-PCR. (b) The expression of miR-876 was detected in different HCC cells by qRT-PCR. (c) The expression of miR-876 was detected in 50 pairs of HCC and their corresponding nontumor samples. *T*: tumor; *N*: nontumor. (d) The percentage of differentially expressed miR-876 in 50 pairs of HCC samples. (e) The expression of miR-876 was detected in HCC samples with or without tumor thrombus. (f) The expression of miR-876 was detected in HCC samples with or without liver cirrhosis.

To explore the potential tumor-related key miRNAs in HCC, we analyzed the miRNA expression data of 49 pairs of tumors and matched adjacent tissues in TCGA public database. As shown in Table 1, the top 20 significantly differentially expressed miRNAs are presented. We mainly focused on the tumor suppressor miRNAs, and the top 6 differentially expressed suppressor miRNAs in Table 1 were miR-4482, miR-4720, miR-490, miR-4648, miR-7849, and miR-876. Of these, we only focused on miR-490 and miR-876 since the rest of the miRNAs mentioned above have barely been reported in any study. We found that both miR-490 and miR-876 were expressed at significantly low levels in SMMC-7721 cells compared with L02 cells, a normal liver cell line, but miR-876 was expressed at lower levels than miR-490 in SMMC-7721 cells (Figure 1(a)). Thus, miR-876 was chosen for further investigation in this study. Further detection revealed that miR-876 was notably expressed at high levels in L02 cells but decreased in all tumor cells, of which, its expression was the highest in SMMC-7721 and lowest in HCC-LM3 cells (Figure 1(b)); therefore, the SMMC-7721 and HCC-LM3 cell lines were chosen for further experiments. Thus, we identified miR-876 from TCGA database and found that it was expressed at low levels in HCC cells.

### 3.2. miR-876 Expression Was Correlated with Clinicopathological Characteristics

We next studied miR-876 expression in clinical HCC tissues, and 50 pairs of HCC tumor and peritumoral tissues were collected for miR-876 detection. As shown in Figures 1(c) and 1(d), miR-876 expression was significantly high in only 11 and low in 31 pairs of samples, and the remaining 8 pairs of samples had similar miR-876 levels. Therefore, we further examined miR-876 expression in 127 HCC samples, and chi-squared analysis revealed that the miR-876 expression level was significantly correlated with liver cirrhosis, tumor thrombus, and TNM stage but was not associated with other parameters such as age, gender, or differentiation (Table 2). To confirm the results, a nonparametric test of liver cirrhosis or tumor thrombus was applied and showed similar results (Figures 1(e) and 1(f)). The results above suggest that miR-876 may play an important role in tumor invasion or liver cirrhosis.

### 3.3. miR-876 Regulated Cell Invasion, EMT, and Collagen Expression

**Figure 2:**
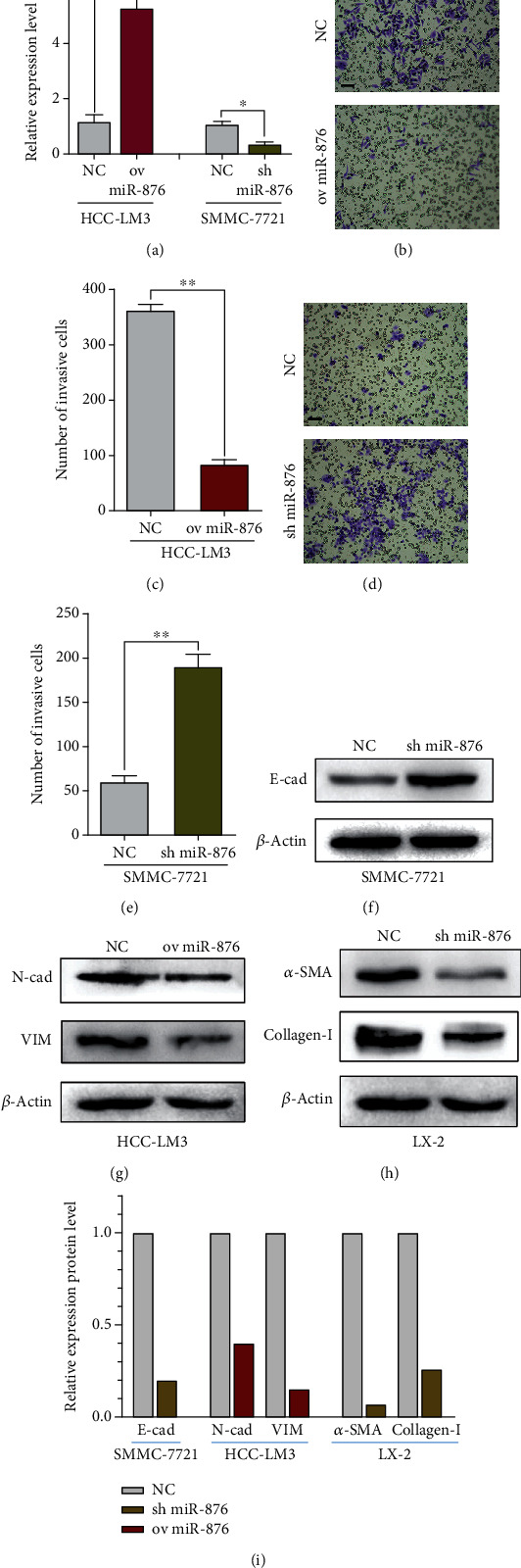
miR-876 regulated cell invasion, EMT, and collagen expression. (a) The expression of miR-876 was detected in indicated treated HCC cells. (b–f) The invasion abilities of indicated treated HCC-LM3 (b, c) or SMMC-7721 (d, e) cells measured by transwell assays. Scale bars = 50 *μ*m. (f) The protein level of E-cadherin was measured by WB assays in indicated treated SMMC-7721 cells. (g) The protein levels of N-cadherin or vimentin were measured by WB assays in indicated treated HCC-LM3 cells. (h) The protein levels of *α*-SMA or collagen-I were measured by WB assays in indicated treated LX-2 cells. (i) The results from Western blot assays of (f–h).

We next examined the cellular functions of miR-876 in HCC cells. miR-876 was upregulated in HCC-LM3 cells and downregulated in SMMC-7721 cells by miR-876 pEX-3 plasmids, and the effectiveness was confirmed by qRT-PCR (Figure 2(a)). Transwell assays revealed that miR-876 upregulation inhibited cell invasion in HCC-LM3 cells, whereas miR-876 downregulation enhanced invasion in SMMC-7721 cells (Figures 2(b)–2(e)). However, miR-876 did not affect the proliferation or apoptosis in HCC cells (data not shown). EMT is a biological process during which epithelial cells lose their epithelial features and acquire mesenchymal features and invasive or metastatic abilities. Therefore, we hypothesized that miR-876 affects invasion via EMT. Western blot (WB) analysis demonstrated that miR-876 overexpression increased E-cadherin expression and decreased N-cadherin and vimentin expression in HCC-LM3 cells, while miR-876 downregulation increased EMT progression in SMMC-7721 cells (Figures 2(f), 2(g), and 2(i)). To verify the role of miR-876 in liver fibrosis, we tested the effects of miR-876 on collagen or *α*-SMA in LX-2 cells. WB results showed that miR-876 inhibition increased the expression of collagen-I or *α*-SMA (Figures 2(g)–2(i)).

### 3.4. miR-876 Targeted POSTN and Inhibited Its Expression

**Figure 3:**
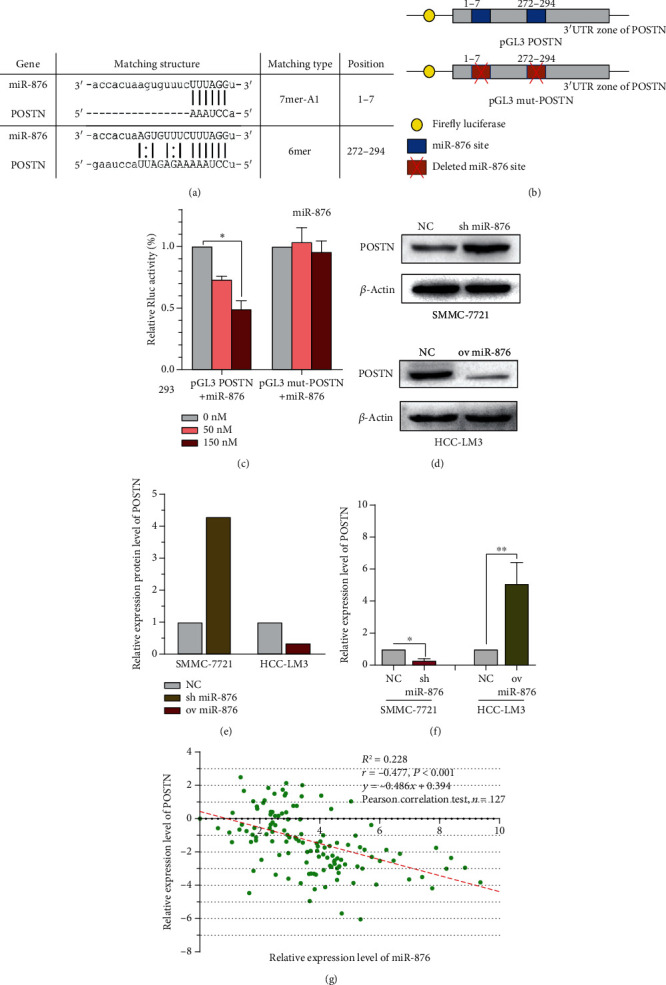
miR-876 regulates the expression of POSTN. (a) The prediction for miR-876 binding sites on POSTN transcript. (b) Schematic outlining the wild-type and mut-POSTN luciferase plasmid. (c) Luciferase activity in HEK-293 cells cotransfected with indicated miR-876 concentration or indicated POSTN luciferase reporter transcript. Data are showed as the ratio of firefly activity to Renilla luciferase activity. (d, e) The protein level of POSTN was measured by WB assays in indicated treated SMMC-7721 or HCC-LM3 cells. (f) The mRNA expression of POSTN was detected in indicated treated SMMC-7721 or HCC-LM3 cells by qRT-PCR. (g) The correlation analysis of POSTN and miR-876 in 127 tissues of HCC patients.

Previous studies have reported that POSTN activates EMT in other tumors [20, 21], and bioinformatics (miRanda) analysis revealed that POSTN has two potential binding sites with miR-876 (Figure 3(a)), thus further confirming the hypothesis. The dual-luciferase reporter assay showed that the pGL3 POSTN+miR-876 group had significantly lower relative luciferase activity compared to the MUT group (Figures 3(b) and 3(c)), suggesting that miR-876 may regulate POSTN. WB analysis revealed that POSTN expression was decreased in miR-876-overexpressing cells, and its expression was increased when miR-876 was inhibited in SMMC-7721 cells (Figures 3(d) and 3(e)), and qRT-PCR showed similar results (Figure 3(f)). We further confirmed the results in clinical HCC samples, and we found that miR-876 was significantly inversely correlated with POSTN, with a correlation coefficient of -0.477 (Figure 3(g)). The above results suggest that miR-876 targets POSTN and inhibits its expression.

### 3.5. miR-876 Inhibited EMT and Collagen via POSTN

**Figure 4:**
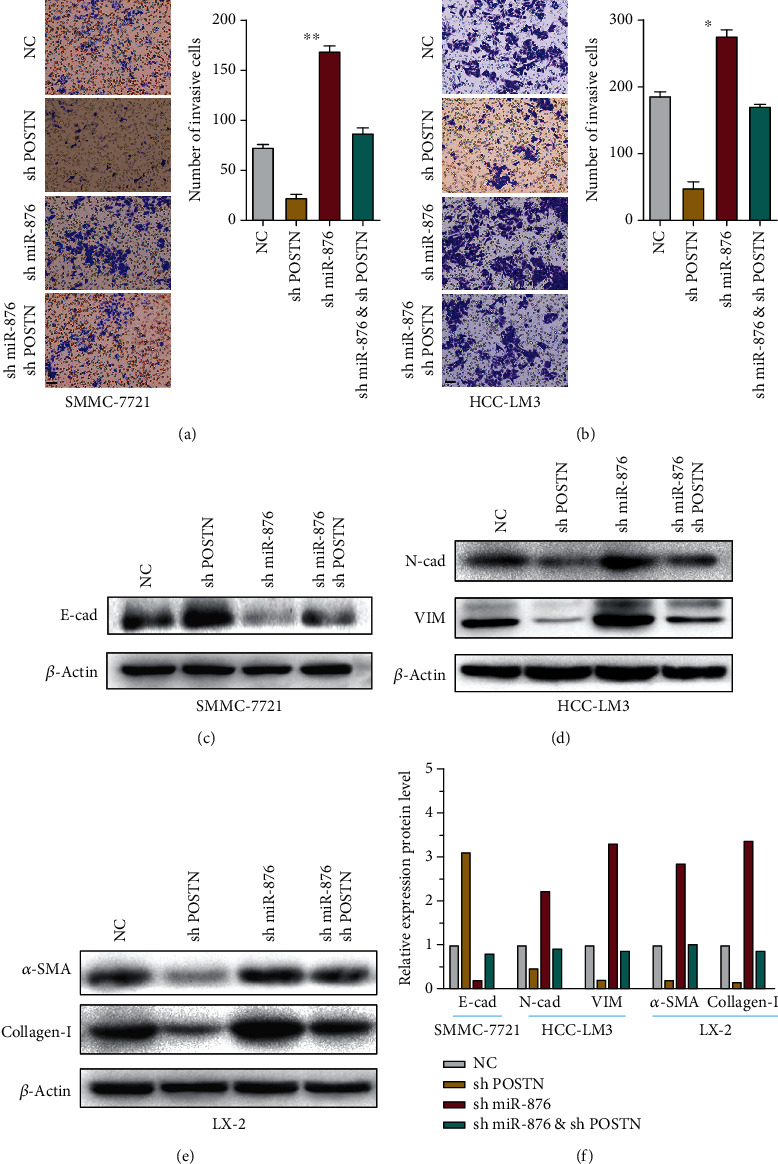
miR-876 inhibited EMT and collagen via POSTN. (a, b) The invasion abilities of indicated treated SMMC-7721 (a) or HCC-LM3 (b) cells measured by transwell assays. Scale bars = 50 *μ*m. (c) The protein level of E-cadherin was measured by WB assays in indicated treated SMMC-7721 cells. (d) The protein levels of N-cadherin or vimentin were measured by WB assays in indicated treated HCC-LM3 cells. (e) The protein levels of *α*-SMA or collagen-I were measured by WB assays in indicated treated LX-2 cells. (f) The results from Western blot assays of (c–e).

We next wondered whether miR-876 functions via POSTN. Transwell assays showed that knockdown of miR-876 increased the number of invasive cells, and the effect was blocked by further POSTN silencing in SMMC-7721 cells (Figure 4(a)). Similar results were found in HCC-LM3 cells (Figure 4(b)). Furthermore, WB results showed that silencing miR-876 enhanced the EMT: the expressions of N-cadherin and vimentin were increased but E-cadherin was decreased, whereas the induction was inhibited by further POSTN knockdown (Figures 4(c), 4(d), and 4(f)). Similarly, collagen-I and *α*-SMA were upregulated by miRNA knockdown and blocked by silencing POSTN in LX-2 cells, as shown in Figures 4(e) and 4(f). Thus, the results above suggest that miR-876 inhibits EMT and collagen via POSTN.

### 3.6. miR-876 Promotes Tumor Progression *In Vivo*

**Figure 5:**
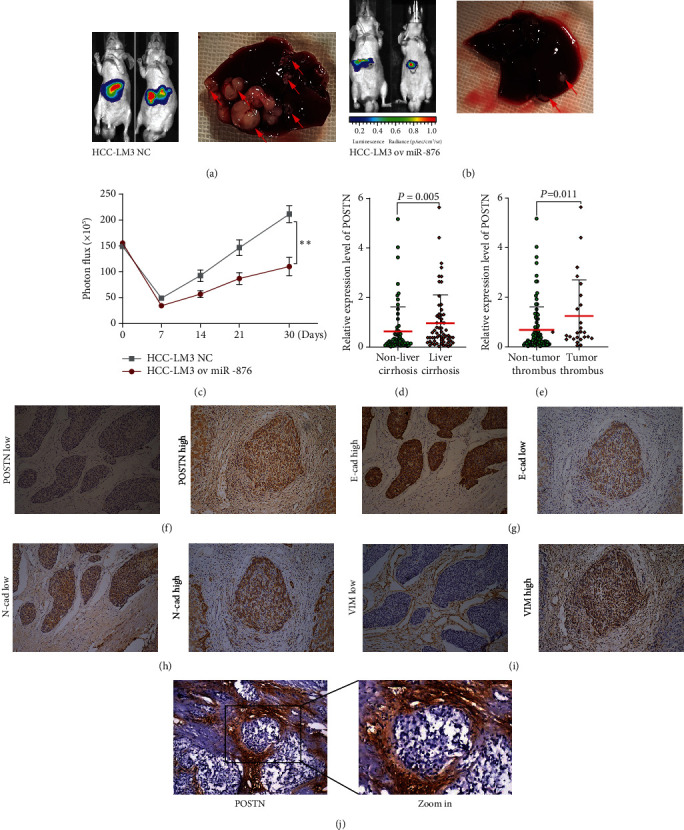
POSTN was associated with EMT and liver cirrhosis in clinical HCC tissues. (a–c) Animal experiments, the luciferase intensities were measured each week (c) after intracapsular injection with NC (a) or ov miR-876 (b) HCC-LM3 cells in the liver, liver cancer in situ (the red arrows point to), were showed by autopsy (a, b). (d) The expression of POSTN was detected in HCC samples with or without tumor thrombus. (e) The expression of POSTN was detected in HCC samples with or without liver cirrhosis. (f–i) Immunohistochemical staining of POSTN and EMT markers in HCC tissues; representative images of POSTN (f), E-cadherin (g), N-cadherin (h), or vimentin (i) immunostaining of low or high, respectively. (j) Representative image of POSTN immunostaining in HCC with liver cirrhosis tissue.

We next studied the role of miR-876 in *in vivo* conditions. HCC-LM3 NC cells (NC) or stable miR-876-overexpressing HCC-LM3 cells were intracapsular injected into the liver of nude mice. The liver cancer of each mouse was analyzed every week by a luciferase IVIS system (Figures 5(a)–5(c)). The *in vivo* imaging results showed that the luciferase intensity of the ov miR-876 group was significantly lower than that of the NC group (Figures 5(a)–5(c)). A month later, the pathological examination results showed that the liver cancer lesions of the ov miR-876 group were obviously much less and smaller than those of the NC group (Figures 5(a) and 5(b)).

### 3.7. POSTN Was Associated with EMT and Liver Cirrhosis in Clinical HCC Tissues

We next confirmed the functions of POSTN in clinical HCC samples. First, we examined POSTN expression by qRT-PCR in 127 samples. As shown in Table 3, the chi-squared analysis revealed that POSTN overexpression was associated with tumor number, liver cirrhosis, tumor thrombus, and TNM stage. A subsequent nonparametric test also showed similar results for liver cirrhosis and tumor thrombus. Furthermore, we wanted to study POSTN expression at the protein level. We detected POSTN expression by IHC in 80 HCC samples. As shown in Figures 5(f)–5(i) and Table 4, POSTN was significantly correlated with N-cadherin and vimentin but inversely associated with E-cadherin. In addition, we found that HCC patients with liver cirrhosis expressed high POSTN (Figure 5(j)).

### 3.8. Coexpression of miR-876 and POSTN May Be a Useful Factor for HCC Survival

**Figure 6:**
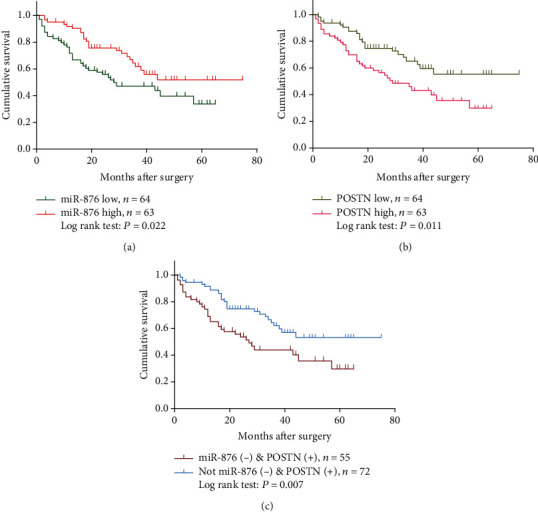
Coexpression of miR-876 and POSTN may be a useful factor for HCC survival. (a) KM survival curves for the overall survival of 127 HCC patients according to the relative expression of miR-876 expression. (b) KM survival curves for the overall survival of 127 HCC patients according to the relative expression of POSTN expression. (c) KM survival curves for the overall survival of 127 HCC patients according to the relative expression of miR-876 and POSTN coexpression.

We next studied the association of miR-876 and POSTN expression with HCC survival. Univariate analysis showed that tumor size, liver cirrhosis, tumor thrombus, TNM stage, miR-876, and POSTN expression are risk factors for HCC overall survival, and KM survival analysis indicated that HCC patients with low miR-876 expression or high POSTN expression had significantly low survival rates (Figures 6(a) and 6(b)). Further multivariate analysis revealed that tumor thrombus and POSTN expression are independent risk factors for survival (Table 5). Furthermore, when we combined miR-876 and POSTN together, patients with miR-876 low and POSTN high expression had a lower overall survival rate than patients analyzed for POSTN alone (Figure 6(c)).

## 4. Discussion

In this study, we screened out miR-876 from TCGA database and confirmed its low expression in HCC cells and samples. Further study revealed that miR-876 regulates EMT and fibrosis via POSTN, thus promoting tumor invasion and metastasis. Finally, we found that aberrant miR-876 or POSTN expression was significantly correlated with HCC prognosis.

Increasing evidence has demonstrated that dysregulated miRNAs have substantial roles in the carcinogenesis, progression, and metastasis of HCC and may serve as meaningful diagnostic or prognostic markers in the clinic [22, 23]. Some miRNAs, such as miR-224 [24], miR-429 [25], miR-17 [26], miR-148a [27], miR-517a [10], and miR-552 [3], act as tumor promoters and are highly expressed in HCC while other miRNAs, such as miR-34a [28], miR-139 [29], miR-135a [30], miR-610 [31], miR-612 [32], and miR-375 [33], are considered tumor suppressors. We screened 49 pairs of tumors and their peritumoral controls in TCGA database and found that miR-876 was expressed at low levels in HCC samples. Furthermore, we tested miR-876 expression in HCC cells and clinical tissues and confirmed its antitumor roles in HCC. Our results are consistent with numerous studies of miR-876 in other tumors, such as breast cancer, lung cancer, cholangiocarcinoma, osteosarcoma, and gastric cancer [34–38]. The roles of miR-876 seem to be diverse in different kinds of tumors. For example, in HCC, miR-876 was reported to affect cell viability and morphology [12]. In gastric cancer, it was revealed to regulate cell apoptosis or proliferation, and in glioma and lung cancer, it suppressed EMT transition [38]. In our study, we found that miR-876 inhibited cell invasion, EMT, and fibrosis, which were somewhat different from other studies. To confirm the results, we first determined the role of miR-876 in an HSC cell line and found that its knockout activated the expression of *α*-SMA and collagen-I in LX-2 cells. We also found that miR-876 expression was correlated with liver cirrhosis. These data imply that miR-876 serves as a tumor suppressor and is a promising predictor of prognosis in HCC.

Our results in this study also suggest that POSTN may be a potential biomarker for HCC invasion or metastasis. Contrary to miR-876, POSTN was increased in HCC tumor cells and clinical tissues. First, we found that the expression of POSTN was higher in HCC-LM3 cells, a tumor cell line with strong metastatic potential, compared to SMMC-7721 cells. Second, the expression of POSTN was significantly inversely correlated with miR-876 expression, which was low in HCC tissues. Finally, survival analysis revealed that patients with high POSTN expression had low overall survival rates. Moreover, although POSTN was viewed as an oncogene in the majority of tumors, numerous studies reported that POSTN was only expressed in stroma, not in solid tumor cells in some tumors. For example, as a tumor promoter, POSTN was only overexpressed in the stroma of prostate cancer, lung cancer, bladder cancer, and colorectal cancer [13, 14]. To confirm the expression and distribution of POSTN in HCC, we further detected the protein level of POSTN by immunohistochemistry. The results showed that POSTN was expressed in both hepatocytes and stroma. However, HCC stroma seems to express higher POSTN than substantial tissue. Our results are consistent with some previous studies of HCC [16, 39]. These data indicated that, apart from its roles in EMT confirmed in this study and plenty of other reports, POSTN may have other roles related to ECM. As expected, we found that POSTN also affected liver cirrhosis. Our data indicated that POSTN promoted the expression of matrix proteins such as *α*-SMA and collagen in hepatic stellate cells, and the clinical correlation analysis revealed that high POSTN was associated with liver cirrhosis. The IHC results also showed that POSTN was strongly expressed in stroma and fibrotic tissues. Our investigation suggests that POSTN may facilitate tumor progression in multiple ways.

Interestingly, we found that both miR-876 and POSTN were correlated with EMT, fibrosis thrombus, and HCC invasion and metastasis. Nevertheless, whether EMT or fibrosis or EMT combined with fibrosis contributes to tumor metastasis appears to be complicated. A large number of studies have investigated and confirmed that the EMT process participates in tumor progression via the transformation into mesenchymal cells with invasive and metastatic potential [40]. Studies discussing the relationship between liver fibrosis and tumor metastasis are relatively rare. Tumor metastasis is a rather complex process with many theoretical hypotheses, with the “seed and soil” hypothesis among the most popular theories [41]. According to this theory, something in the microenvironment of the metastatic sites must support metastatic colonization to form this specific microenvironment. Indeed, disruption of ECM homeostasis is thought to provide the main extrinsic drivers of tumor progression [42]. Fibrosis is a common disruption of the delicate balance of ECM homeostasis [43]. For example, studies have shown that fibrotic disorders of the lung or breast lead to tumor progression [44, 45]. Thus, tumors may appropriate sites of future metastasis before their arrival in a manner that resembles the development of tissue fibrosis. In this study, we found that miR-876 and POSTN are also associated with liver fibrosis, and overexpression of POSTN increased the expression of collagen and *α*-SMA. This finding is consistent with other previous studies. For example, Kumar et al. [46] found that POSTN induced liver fibrogenesis by activating lysyl oxidase, and Amara et al. [47] reported that POSTN mediated collagen deposition in the liver. Interestingly, we also found that miR-876 or POSTN was closely correlated to tumor thrombus, which arises from the vascular invasion and is an important biological feature of HCC [48]. Tumor thrombus is a special type of intrahepatic metastasis of HCC [49] and is one of the main reasons for the poor prognosis of HCC. Therefore, we hypothesized that miR-876 and POSTN may enhance the invasion of tumor cells in situ via EMT and disrupt ECM homeostasis via fibrogenesis to form a favorable metastatic microenvironment, thus inducing tumor progression and poor prognosis.

## 5. Conclusions

In summary, this study identified miR-876 as an important miRNA in HCC and demonstrated that miR-876 inhibited cell invasion and fibrosis by negatively regulating POSTN. Further research revealed that miR-876 and POSTN were inversely correlated in HCC samples and associated with EMT, liver cirrhosis, and tumor invasion. miR-876 and POSTN may be potential therapeutic targets of HCC.

## Figures and Tables

**Table 1:** Top 20 dysregulated miRNAs from the HCC TCGA database.

Upregulated miRNAs			Downregulated miRNAs		
Rank	miRNA	Fold changes (*N*/*T*)	Rank	miRNA	Fold changes (*N*/*T*)
1	hsa-mir-424	0.26	1	hsa-mir-4482	13.96
2	hsa-mir-139	0.27	2	hsa-mir-490	13.05
3	hsa-mir-199a-1	0.35	3	hsa-mir-4720	12.55
4	hsa-mir-199a-2	0.35	4	hsa-mir-4686	12.13
5	hsa-mir-199b	0.36	5	hsa-mir-7849	11.54
6	hsa-mir-142	0.43	6	hsa-mir-876	11.11
7	hsa-mir-144	0.44	7	hsa-mir-5702	10.81
8	hsa-mir-451a	0.44	8	hsa-mir-1264	9.56
9	hsa-mir-542	0.45	9	hsa-mir-3182	9.03
10	hsa-mir-223	0.46	10	hsa-mir-873	8.53
11	hsa-mir-3607	0.46	11	hsa-mir-1258	8.29
12	hsa-mir-101-1	0.46	12	hsa-mir-4799	7.88
13	hsa-mir-101-2	0.46	13	hsa-mir-3166	7.69
14	hsa-mir-145	0.48	14	hsa-mir-6729	7.65
15	hsa-let-7c	0.50	15	hsa-mir-4679-2	7.05
16	hsa-mir-10a	0.50	16	hsa-mir-4710	6.98
17	hsa-mir-99a	0.51	17	hsa-mir-1193	6.62
18	hsa-mir-125b-1	0.51	18	hsa-mir-4760	6.35
19	hsa-mir-125b-2	0.52	19	hsa-mir-1225	6.15
20	hsa-mir-150	0.55	20	hsa-mir-3616	6.06

*N*: nontumor; *T*: tumor.

**Table 2:** Clinical characteristics and expressions of miR-876 in 127 HCC patients.

Parameters	Total case	miR-876		
		High	Low	*P* value
All case	127	63	64	
Gender				0.610
Male	22	12	10	
Female	105	51	54	
Age (years)				0.129
≥60	25	9	16	
<60	102	54	48	
Tumor size (cm)				0.135
>5	96	44	52	
≤5	31	19	12	
Tumor number				0.728
Multiple	34	16	18	
Single	93	47	46	
HBsAg				0.101
Yes	111	52	59	
No	16	11	5	
AFP				0.535
>400	64	30	34	
≤400	63	33	30	
Liver cirrhosis				0.026
Yes	61	24	37	
No	66	39	27	
Child stage				0.324^∗^
A	118	57	61	
B	9	6	3	
Tumor thrombus				0.031
Yes	26	8	18	
No	101	55	46	
Stage (UICC)				0.021
I-II	78	45	33	
III-IV	49	18	31	

^∗^Fisher's exact test.

**Table 3:** Clinical characteristics and expressions of POSTN in 127 HCC patients.

Parameters	Total case	POSTN		
		High	Low	*P* value
All case	127	63	64	
Gender				0.370
Male	22	9	13	
Female	105	54	51	
Age (years)				0.789
≥60	25	13	12	
<60	102	50	52	
Tumor size (cm)				0.163
>5	96	51	45	
≤5	31	12	19	
Tumor number				0.040
Multiple	34	22	12	
Single	93	41	52	
HBsAg				0.116
Yes	111	58	53	
No	16	5	11	
AFP				0.424
>400	64	34	30	
≤400	63	29	34	
Liver cirrhosis				0.002
Yes	61	39	22	
No	66	24	42	
Child stage				1.000^∗^
A	118	59	59	
B	9	4	5	
Tumor thrombus				0.007
Yes	26	19	7	
No	101	44	57	
Stage (UICC)				0.015
I-II	78	32	46	
III-IV	49	31	18	

^∗^Fisher's exact test.

**Table 4:** Correlations between POSTN and EMT markers in 80 HCC tissues via IHC.

		E-cadherin			N-cadherin			Vimentin		
		Low	High	*P* value	Low	High	*P* value	Low	High	*P* value
POSTN	Low	27	28		42	33		41	14	
	High	19	6		8	17		7	18	
	*P* value			0.024			0.038			0.001

**Table 5:** Univariate and multivariate survival analyses of the prognostic factors associated with survival in HCC patients (*n* = 127).

OS	Univariate analysis			Multivariate analyses		
	Patients/*n*	Mean survival time	*P* value	HR	95% CI	*P* value
Gender						
Male/female	105/22	38/45	0.368			
Age (years)						
≥60/<60	25/102	38/45	0.698			
Tumor size (cm)						
≤5/>5	31/96	53/36	0.037			0.756
Tumor number						
Single/multiple	93/34	39/47	0.494			
HBsAg						
Yes/no	111/16	43/44	0.303			
AFP						
>400/≤400	64/63	37/47	0.334	2.530	1.433-4.468	0.001
Liver cirrhosis						
Yes/no	61/66	38/44	0.041			0.480
Child stage						
A/B	118/9	44/28	0.489			
Tumor thrombus						
Yes/no	26/101	23/48	<0.001			0.205
Stage (UICC)						
I-II/III-IV	78/49	50/30	0.001			0.386
miR-876 expression						
Low/high	64/63	35/50	0.022			0.496
POSTN expression						
Low/high	64/63	51/34	0.012	1.717	1.008-2.926	0.047

## Data Availability

Research data of the paper is not applicable as the clinical data include legal and ethical concerns.

## Abbreviations

HCC: Hepatocellular carcinoma
miRNA: microRNA
3′ UTR: 3′ untranslated region
CCA: Cholangiocarcinoma
POSTN: Periostin
ECM: Extracellular matrix
IHC: Immunohistochemistry
TCGA: The Cancer Genome Atlas.

## Supplementary Materials

Supplementary Materials Table S1: sample IDs used in the study from TCGA database. Table S2: primer sequences used in this study.
