# Erratum to “miR-876 Inhibits EMT and Liver Fibrosis via POSTN to Suppress Metastasis in Hepatocellular Carcinoma”

**DOI:** 10.1155/2020/7043985

**Published:** 2020-12-12

**Authors:** Kai Chen, Zhonghu Li, Mengyun Zhang, Bo Wang, Tao Peng, Yanbing Shen, Jianxin Zhang, Jiaxin Ye, Yu Liu, Di Tang, Minjie Peng, Dandan Ma, Zhengkang Xiao, Yujun Zhang, Weidong Jin, Xiaowu Li

**Affiliations:** ^1^Hepatobiliary Surgery Institute, Southwest Hospital, Army Medical University, China; ^2^Department Hepatobiliary Surgery Institute, Chengdu Fifth People's Hospital, China; ^3^Department General Surgery, Central Theater Command General Hospital of PLA, China; ^4^Department Rheumatology of Integrated Traditional Chinese and Western Medicine, Central Theater Command General Hospital of PLA, China; ^5^Department of Hepatobiliary Surgery, The First Affiliated Hospital of Yangtze University, Jingzhou, China; ^6^Hepatobiliary Surgery & Carson International Cancer Shenzhen University General Hospital & Shenzhen University Clinical Medical Academy Center, Shenzhen University, China

In the article titled “miR-876 Inhibits EMT and Liver Fibrosis via POSTN to Suppress Metastasis in Hepatocellular Carcinoma” [1], information was omitted from the Acknowledgments section. The corrected Acknowledgments section is shown below. This mistake was introduced during the production of the article, and the publisher apologizes for this error.
