# Supplementary material for: miR-876 Inhibits EMT and Liver Fibrosis via POSTN to Suppress Metastasis in Hepatocellular Carcinoma
Source: Biomed Res Int. 2020 Oct 5;2020:1964219. doi: 10.1155/2020/1964219 (PMC7559219; doi:10.1155/2020/1964219)
Supplement: Supplementary Materials — Table S1: sample IDs used in the study from TCGA database. Table S2: primer sequences used in this study. [file 1964219.f1.pdf]

Table S1 Sample IDs used in the study from TCGA database

Group	Sample ID	Group	Sample ID
Peritumoral samples	TCGA-BC-A10Q-11	Tumor	TCGA-BC-A10Q-01
	TCGA-BC-A10R-11		TCGA-BC-A10R-01
	TCGA-BC-A10T-11		TCGA-BC-A10T-01
	TCGA-BC-A10U-11		TCGA-BC-A10U-01
	TCGA-BC-A10W-11		TCGA-BC-A10W-01
	TCGA-BC-A10X-11		TCGA-BC-A10X-01
	TCGA-BC-A10Y-11		TCGA-BC-A10Y-01
	TCGA-BC-A10Z-11		TCGA-BC-A10Z-01
	TCGA-BC-A110-11		TCGA-BC-A110-01
	TCGA-BC-A216-11		TCGA-BC-A216-01
	TCGA-BD-A2L6-11		TCGA-BD-A2L6-01
	TCGA-BD-A3EP-11		TCGA-BD-A3EP-01
	TCGA-DD-A113-11		TCGA-DD-A113-01
	TCGA-DD-A114-11		TCGA-DD-A114-01
	TCGA-DD-A116-11		TCGA-DD-A116-01
	TCGA-DD-A118-11		TCGA-DD-A118-01
	TCGA-DD-A119-11		TCGA-DD-A119-01
	TCGA-DD-A11A-11		TCGA-DD-A11A-01
	TCGA-DD-A11B-11		TCGA-DD-A11B-01
	TCGA-DD-A11C-11		TCGA-DD-A11C-01
	TCGA-DD-A11D-11		TCGA-DD-A11D-01
	TCGA-DD-A1EB-11		TCGA-DD-A1EB-01
	TCGA-DD-A1EC-11		TCGA-DD-A1EC-01
	TCGA-DD-A1EE-11		TCGA-DD-A1EE-01
	TCGA-DD-A1EG-11		TCGA-DD-A1EG-01
	TCGA-DD-A1EH-11		TCGA-DD-A1EH-01
	TCGA-DD-A1EI-11		TCGA-DD-A1EI-01
	TCGA-DD-A1EJ-11		TCGA-DD-A1EJ-01
	TCGA-DD-A1EL-11		TCGA-DD-A1EL-01
	TCGA-DD-A39V-11		TCGA-DD-A39V-01
	TCGA-DD-A39W-11		TCGA-DD-A39W-01
	TCGA-DD-A39X-11		TCGA-DD-A39X-01
	TCGA-DD-A39Z-11		TCGA-DD-A39Z-01
	TCGA-DD-A3A1-11		TCGA-DD-A3A1-01
	TCGA-DD-A3A2-11		TCGA-DD-A3A2-01
	TCGA-DD-A3A3-11		TCGA-DD-A3A3-01
	TCGA-DD-A3A4-11		TCGA-DD-A3A4-01
	TCGA-DD-A3A5-11		TCGA-DD-A3A5-01
	TCGA-DD-A3A6-11		TCGA-DD-A3A6-01
	TCGA-DD-A3A8-11		TCGA-DD-A3A8-01
	TCGA-EP-A12J-11		TCGA-EP-A12J-01
	TCGA-EP-A26S-11		TCGA-EP-A26S-01

	TCGA-EP-A3RK-11	TCGA-EP-A3RK-01
	TCGA-ES-A2HT-11	TCGA-ES-A2HT-01
	TCGA-FV-A23B-11	TCGA-FV-A23B-01
	TCGA-FV-A2QR-11	TCGA-FV-A2QR-01
	TCGA-FV-A3I0-11	TCGA-FV-A3I0-01
	TCGA-FV-A3I1-11	TCGA-FV-A3I1-01
	TCGA-G3-A3CH-11	TCGA-G3-A3CH-01

Table S2. Primers sequences used in this study

Gene	Primer	Sequence
U6	Forward	CTCGCTTCGGCAGCACA
	Backward	AACGCTTCACGAATTTGCGT
miR-876-5P	Forward	GGGGTGGATTTCTTTGTGAATC
miR-490-3P	Forward	CAACCTGGAGGACTCCATG
POSTN	Forward	GCGAGATCATCAAGCCAGCAGAG
	Backward	ATGTCCAGTCTCCAGGTTGTGTCA
shPOSTN	Sequence	CCGGCGGTGACAGTATAACAGTAAACTC<br>GAGTTTACTGTTATACTGTCACCGTTTTTG
beta-actin	Forward	GCGGACTATGACTTAGTTGCGTTACA
	Backward	TGCTGTCACCTTCACCGTTCCA
miR-876 mimics	sense	UGGAUUUCUUUGUGAAUCACCA
	antisense	GUGAUUCACAAAGAAAUCCAUU
mimic control	sense	UUCUCCGAACGUGUCACGUTT
	antisense	ACGUGACACGUUCGGAGA
miR-876 inhibitor	Sequence	UGGUGAUUCACA AAGAAAUCCA
Inhibitor NC	Sequence	CAGUACUUUUGUGUAGUACAA
